# Policies on Conflicts of Interest in Health Care Guideline Development: A Cross-Sectional Analysis

**DOI:** 10.1371/journal.pone.0166485

**Published:** 2016-11-15

**Authors:** Cristina Morciano, Vittorio Basevi, Carla Faralli, Michele Hilton Boon, Sabina Tonon, Domenica Taruscio

**Affiliations:** 1 Centro Nazionale Malattie Rare, Istituto Superiore di Sanità, Rome, Italy; 2 Centro di Documentazione sulla Salute Perinatale e Riproduttiva, Servizio assistenza distrettuale, medicina generale, pianificazione e sviluppo dei servizi sanitari, Regione Emilia-Romagna, Bologna, Italy; 3 Servizio Informatico, Documentazione, Biblioteca e Attività Editoriali, Istituto Superiore di Sanità, Rome, Italy; 4 MRC/CSO Social and Public Health Sciences Unit, University of Glasgow, Glasgow, United Kingdom; 5 Formerly Centro Nazionale Malattie Rare, Istituto Superiore di Sanità, Rome, Italy; York University, CANADA

## Abstract

**Objective:**

To assess whether organisations that develop health care guidelines have conflict of interest (COI) policies and to review the content of the available COI policies.

**Methods:**

Survey and content analysis of COI policies available in English, French, Spanish, and Italian conducted between September 2014 and June 2015. A 24-item data abstraction instrument was created on the basis of guideline development standards.

**Results:**

The survey identified 29 organisations from 19 countries that met the inclusion criteria. From these organisations, 19 policies were eligible for inclusion in the content analysis. Over one-third of the policies (7/19, 37%) did not report or did not clearly report whether disclosure was a prerequisite for membership of the guideline panel. Strategies for the prevention of COI such as divestment were mentioned by only two organisations. Only 21% of policies (4/19) used criteria to determine whether an interest constitutes a COI and to assess the severity of the risk imposed.

**Conclusions:**

The finding that some organisations, in contradiction of widely available standards, still do not have COI policies publicly available is concerning. Also troubling were the findings that some policies did not clearly report critical steps in obtaining, managing and communicating disclosure of relationships of interest. This in addition to the variability encountered in content and accessibility of COI policies may cause confusion and distrust among guideline users. It is in the interest of guideline users and developers to design an agreed-upon, comprehensive, clear, and accessible COI policy.

## Introduction

The problem of conflict of interest (COI) in health care guidelines has received growing attention from health care guideline developers and users in recent years. Inadequate management of COI can result in bias and have important implications for public confidence in both the guidelines and the organisations that produce them [1–5]. In response to this problem, numerous recommendations for improving the identification and management of COI in guideline development have been issued [6–12]. The U.S. Institute of Medicine (IOM) and Guidelines International Network (G-I-N), a global network of individuals and organisations interested in guidelines [www.g-i-n.net/about-g-i-n], have both produced standards for creating trustworthy guidelines [13–16] which include guiding principles to identify and manage COI. The importance of recording and addressing COI is also reflected in the appraisal criteria of AGREE II [17], an international tool for assessing the quality and reporting of health care guidelines.

Research on COI in guidelines has focused mainly on compliance with IOM and AGREE II standards [18–21] as well as on the prevalence of COI among members of guideline development groups [20,22–29]. Findings suggest that adherence to these standards is poor [18–21] and COI are both common [19,20,23,25–29] and rarely disclosed [23,26,27,29].

For guideline developers, a first step in addressing COI is to have relevant policies and procedures in place. To our knowledge only one previous study [30] has surveyed and examined the content of the COI policies of guideline developers. This cross-sectional study of 37 organisations producing a ‘large’ number of guidelines (defined as five or more guidelines listed in the National Guidelines Clearinghouse in 2009–2010) compared COI policies to IOM standards. Only 17 (17/37, 46%) of the guideline developers studied had a COI policy for guidelines in place and not one policy adhered to all 7 relevant IOM standards [14]. The study was limited to English-language guideline developers and nearly two-thirds of these organisations (24/37, 65%) were in the United States, with the remainder representing four other countries: Canada (5), the United Kingdom (5), New Zealand (2), and the Netherlands (1).

This study expands upon previous research to examine the COI policies of an international sample that includes non-English-language guideline developers.

### Objectives

The aim of our study was to assess whether and how organisations address COI issues related to guideline development, and to identify and quantify problematic areas of underreporting.

We set out to capture a cross-sectional sample of worldwide guideline development organisations and to examine the content of their policies on COI available in English, French, Spanish and Italian through a predefined data abstraction instrument.

## Methods

### Selection of guideline development organisations

Between September 2014 and June 2015 we conducted an Internet search and content analysis of the COI policies of organisations who have a programme of health care guideline development covering a wide range of health topics i.e. a programme not limited to a particular disease or condition or to group or subgroup of diseases or conditions.

We obtained a preliminary list of 53 organisations by searching in the G-I-N database (http://www.g-i-n.net/membership/members-around-the-world) filtered by “activities” and “guideline development” (accessed September 25, 2014).

This list was integrated with a list of organisations obtained through an evidence inventory [31] of surveys of guideline developer organisations. We performed the evidence inventory to list what surveys were available on guideline developer organisations. Studies were sought from MEDLINE (1 January 2009 to 1 August 2014) and limited to English language (S1 Table). The search identified 217 articles. One author (CM) screened the titles and abstracts of all retrieved articles. A survey of European guideline developers was found and included [32] with one companion report [33]. We also included an article [34] which listed guideline handbooks/manuals to identify further guideline developers.

From these studies 60 additional organisations were identified, of which 21 were duplicates, leaving a sample of 92 organisations (S2 Table). In order to identify a manageable subsample of organisations that had the potential to produce guidelines with the most wide-ranging impacts on health, we pragmatically restricted our sample further to exclude organisations whose guideline programmes are limited to single disease areas or single health professions.

### Identification of COI policies

We searched the websites of included organisations for documents that described the guideline development process (manuals, handbooks, methodology articles, webpages), disclosure of relationships of interest (DOI) forms, and any other available documents that mentioned COI issues related to guideline development and available in English, French, Italian or Spanish, with no date restrictions. We did not contact organisations directly to obtain additional information, but used only information publicly available.

We aimed to include documents that addressed COI in guideline development and that provided data for at least two of the items included in each of the following overarching items of the data abstraction instrument: “Information required for disclosure of financial and nonfinancial relationships” and “Procedures for collecting, reviewing disclosure of relationships of interest and managing conflict of interest” (Table 1). We called a document meeting these criteria a “COI policy”. In the content analysis we distinguished the term “relationship (financial and nonfinancial) of interest” and “conflict of interest”. The term “relationship of interest” refers to any interest or activity requested to be declared that may be judged to constitute a COI. Consequently we used the term “disclosure of relationships of interest” to indicate the step that precedes the review of DOI.

**Table 1:** Data abstraction instrument.

**General**
Name of organisation
Country
Type of organisation
Document on guideline development
Conflict of interest policy presence
Date of conflict of interest policy
Source of conflict of interest policy (handbooks/methodological articles/webpages)
Definition(s) of conflict of interest
**Information required for disclosure of financial and nonfinancial relationships**
Types of financial relationships
Financial threshold considered
Types of nonfinancial relationships
Relevance to the guideline topic/issue of the guideline considered
Time period for disclosure considered
About whom is disclosure of relationships of interest collected
Information on financial and nonfinancial relationships of the individual’s personal relationships considered
**Procedures for collecting, reviewing disclosure of relationships of interest and managing conflict of interest**
Disclosure of relationships of interest required prior to selection of the guideline development group
Who reviews disclosure of relationships of interest and make decision
Assessment of risk performed
Divestment required prior to selection of the guideline development group
Exclusion procedure applied
Relationship prohibited
Reported penalties for non-disclosure
**Processes for recording and making publicly accessible disclosure of relationships of interest**
Description of the process to record disclosure of relationships of interest
Completed disclosure forms publicly accessible

### Data extraction and analysis

We developed a 24-item data abstraction instrument (Table 1) based on recommendations and standards of COI proposed by the IOM [13,14] and G-I-N [15]. The types of financial relationships and nonfinancial relationships considered in this study were based on those listed by IOM [13,14] and G-I-N [15].

One author (CM) abstracted information on each organisation and each COI policy into the predefined template and those data were checked by a second author (CF, ST). When there was disagreement regarding the content of a particular COI policy this was resolved by discussion. If the policy was updated during the period of data collection, we updated the abstracted information. Where disclosure forms were publicly available, data from these were integrated with the data extracted from the policies. Where inconsistencies were found, the information in the policy prevailed.

## Results

Twenty-six organisations were excluded from the 92 identified as their remit was restricted to single disease specialties or single professional groups (S2 Table). Of the 66 remaining organisations, 11 were subsequently excluded because they did not provide documents on guideline development in English, French, Italian, or Spanish; 15 because they did not provide publicly accessible documents on guideline development or COI issue guideline related; 5 because their website was not found or not accessible and 6 because they were not guideline developers.

**Fig 1:**
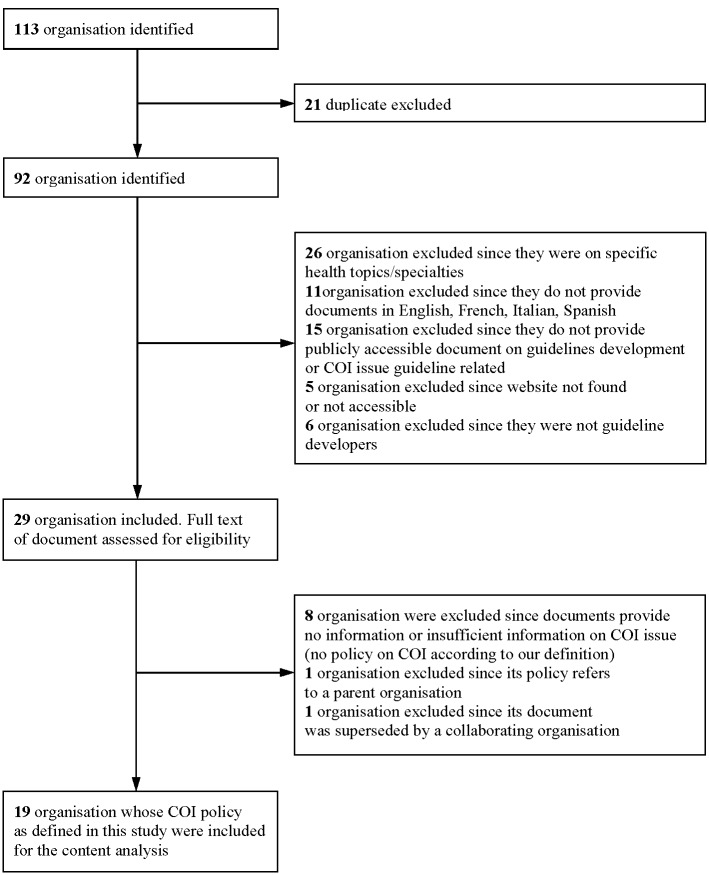
Organisation identification and inclusion criteria.

The documents of the remaining 29 organisations (Table 2) were assessed for the presence of a COI policy according to our definition. Of these organisations 21 were governmental, 4 were not for profit and 4 were professional associations. Ten organisations (10/29, 34%) were excluded from content analysis for the following reasons: 8 because their documents did not mention or provided insufficient information on COI, 1 because its handbook referred to an included parent organisation’s COI policy and 1 because its handbook was superseded by the handbook of its collaborating organisation. In the end 19 COI policies were included in the content analysis from 13 countries and 1 intergovernmental organisation. Fig 1 provides a description of the inclusion of organisations at each stage.

**Table 2:** Characteristics of the included organizations (N = 29).

Organisation	Country	Type of organisation	Document on guideline development	COI policy	Date of COI policy	Source of COI policy
American College of Physicians (ACP)	USA	Professional	Yes [35]	Yes	2010	Science journal [35]
Belgian Health Care Knowledge Centre/Federal Centre of Health Care Expertise (KCE)	Belgium	Government	Yes [36]	No	Not applicable	Not applicable
Canadian Task Force on Preventive Health Care (CTFPHC)	Canada	Government	Yes [37]	Yes	2014	Handbook [37]
US Centers for Disease Control and Prevention Healthcare- Infection Control Practices Advisory Committee (CDC-HICPAC)	USA	Government	Yes [38]	No	Not applicable	Not applicable
US Centers for Disease Control and Prevention Advisory Committee on Immunization Practices (CDC-ACIP)	USA	Government	Yes [39,40]	Yes	2009	Science journal [40]
Centro National de Excelentia Tecnólogica en Salud (CENETEC)	Mexico	Government	Yes [41]	Yes	2007	Handbook [41]
Conseil Scientifique du Domaine de la Santé (CSDS)	Luxembourg	Government	Yes [42]	No	Not applicable	Not applicable
Current Care Guidelines /The Finnish Medical Society Duodecim (FMSD)	Finland	Professional	Yes [43]	Yes	2014	Webpage (flow chart) [43]
Instituto de Evaluación Tecnológica en Salud (IETS)	Colombia	Not for profit	Yes [44]	Yes	2014	Handbook [44]
German Association of the Scientific Medical Societies (AWMF) (Arbeitsgemeinschaft der Wissenschaftlichen Medizinischen Fachgesellschaften)	Germany	Professional	Yes [45]	Yes	2012	Handbook [45]
German Agency for Quality in Medicine (AQuMed ÄZQ) (Das Ärztliche Zentrum für Qualität in der Medizin)	Germany	Professional	Yes [46]	Yes (referred to AWMF) [45]	Not applicable	Not applicable
GuíaSalud (GS)	Spain	Government	Yes [47]	Yes	2007	Handbook [47]
Haute Autorité de Santé (HAS)	France	Government	Yes [48,49]	Yes	2013	Stand alone document posted in the website; other related information in the guideline handbook [48,49]
Kaiser Permanente (KP)	USA	Not for profit	Yes [50]	No	Not applicable	Not applicable
Ministerio de Salud (MS)	Peru	Government	Yes [51]	No	Not applicable	Not applicable
Ministerio de Salud y Protección Social (MSPS)	Colombia	Government	Yes [52]	Yes (superseded by handbook of IETS as collaborating organisation [44])	Not applicable	Not applicable
National Board of Health and Welfare (SS) (Socialstyrelsen)	Sweden	Government	Yes [53]	No	Not applicable	Not applicable
King Saud bin Abdulaziz University for Health Sciences, National and Gulf Center for Evidence Based Health Practice (EBHP)	Saudi Arabia	Government	Yes [54]	No	Not applicable	Not applicable
The National Clinical Effectiveness Committee (NCEC)	Ireland	Government	Yes [55,56]	Yes	2013	Stand alone document posted in the website [56]
National Health and Medical Research Council (NHRMC)	Australia	Government	Yes [57]	Yes	2012	Stand alone document posted in the website [57]
National Institute for Health and Care Excellence (NICE)	UK-England	Government	Yes [58,59]	Yes	2014	Stand alone document posted in the website; other related information in the guideline handbook [58,59]
National Institute of Quality and Innovation (NIKI) (Národný Inštitút Kvality a Inovácií)	Slowakia	Not for profit	Yes [60]	No	Not applicable	Not applicable
Scottish Intercollegiate Guidelines Network (SIGN)	UK-Scotland	Government	Yes [61,62]	Yes	2014	Stand alone document posted in the website; other related information in the guideline handbook [61,62]
Sistema Nazionale Linee Guida (SNLG)	Italy	Government	Yes [63]	Yes	2004	Handbook [63]
Therapeutic Guidelines Limited (TGL)	Australia	Not for profit	Yes [64]	Yes	2014	Stand alone document posted in the website [64]
Universidad Nacional de Colombia, Instituto de Investigaciones Clinicas, Facultad de Medicina (GETS)	Colombia	Government	Yes [65]	Yes	Not reported	Handbook [65]
University of Tartu Medical Faculty, Estonian Health Insurance Foundation, World Health Organization (HE-TU)	Estonia	Government	Yes [66]	Yes	2011	Handbook [66]
US Preventive Services Task Force (USPSTF)	USA	Government	Yes [67]	Yes	2008	Handbook [67]
World Health Organization (WHO)	Intergovernmental	Government	Yes [68]	Yes	2014	Handbook [68]

### Conflict of interest policies

The 19 COI policies were accessible in various formats. Many organisations included their policy in a guideline handbook [37,41,44,45,47,63,65–68] (10/19, 53%). Six organisations [48,56,57,59,61,64] (6/19, 32%) posted their COI policies on their website as a stand alone document. Three of these provided additional information about management of COI in their guideline handbook [49,58,62] with reference to the COI policy posted in the website. The remainder of the organisations included information on COI policy on a webpage as a flow chart [43] or in a methodological article posted on the organisation’s website [35,40] (Table 2).

The date of the COI policy was determined by considering the date of the document itself if published as stand alone document or of the corresponding date of the handbook/manual, methodological article, or webpage where the COI policy was included. The date of the 18 policies for which a date could be determined ranged from 2007 to 2014. Of the 18 policies 7 were published in 2014 (7/18, 39%), 5 were dated between 2011–2013 (5/18, 28%) and 6 were dated prior 2011 (6/18, 33%). Around one third of the policies (Table 3) [37,40,41,43,63,67] (6/19, 32%) did not report a definition of the term “conflict of interest” and the remaining policies provided heterogeneous definitions.

**Table 3:** Definition of conflict of interest by organisation.

Organisation	Definition of conflict of interest
ACP [35]	“Potential financial or nonfinancial conflicts of interest that refer to relationships that a reasonable reader of a guideline would wish to know about and that if not disclosed could compromise the interpretation of the ACP guideline”
CTFPHC [37]	Not reported
CDC-ACIP [39,40]	Not reported
CENETEC [41]	Not reported
FMSD [43]	Not reported
IETS [44]	The activities that may create potential conflicts of interest occur in those circumstances where professional judgment on a primary interest, such as patient’s welfare or the validity of research, may be influenced by a secondary interest, such as financial gain, prestige, personal or professional advancement
AWMF [45]	Not reported
GS [47]	The activities that may create potential conflicts of interest occur in those circumstances where professional judgment on a primary interest, such as patient’s welfare or the validity of research, may be influenced by a secondary interest, such as financial gain, prestige, personal or professional advancement [69]
HAS [48,49]	The relationships of interests may give rise to the conflict of interest. These are therefore two distinct concepts. A conflict of interest arises from a situation in which the relationships of interest of a person may affect, by their nature or intensity, his impartiality or independence in the exercise of his mission regarding the matter under discussion
NCEC [55,56]	“In the context of the work of the NCEC, a COI is any interest that could result in bias in the work or decision making processes of the NCEC”
NHMRC [57]	“Financial interests: an interest must be declared as a potential conflict when benefits or losses either in money or in kind have occurred or may occur at a level that might reasonably be perceived to affect a person’s judgment in relation to a fair decision about evidence and their participation in group decision making”. “Other relationship: an interest must be declared as a potential conflict when a strong position or prejudice or familial connection or other relationship held by a person could reasonably, or be perceived to, affect a person’s judgment in relation to fair decision about evidence and their participation in group decision-making including making an effort to arrive at a consensus”
NICE [58,59]	“A conflict of interest arises when the judgment of someone involved in the work of NICE may be compromised, by the financial or other considerations set out in this policy”
SIGN [61,62]	“Competing interests are defined as any interest of the person, their partners or close relatives (personal) or their department/employer/business (non-personal) which may potentially influence the content, including recommendations, of SIGN guidelines”
SNLG [63]	Not reported
TGL [64]	“Interest statements must comprise a declaration of any interests that may be capable of influencing advice or decisions relating to the operation or activities of TGL, or that may affect the integrity and reputation of TGL”
GETS [65]	The conflict of interest arises when an individual or organisation considers alternatives where interests or benefits coexist and there is a duality of commitment [70]. The conflict is evident when the option that provides personal benefits is selected at the detriment of the option more adherent to ethical principles and general interest
HE-TU [66]	“Any interest which may affect or may reasonably be perceived to affect, the expert objectivity and independence”
USPSTF [67]	Not reported
WHO [68]	“A conflict of interest is a set of circumstances that creates a risk that professional judgment or actions regarding a primary interest will be unduly influenced by a secondary interest” [14]. “Any interest declared by an expert that may affect or reasonably be perceived to affect the expert’s objectivity and independence in providing advice to WHO”

### Disclosure of relationships of interest

All included policies outlined categories of financial relationships but characterization of specific types of nonfinancial relationships was lacking in some cases [39,40,43,47,63] (4/19, 21%) (Table 4). Policies varied in the categories of information that need to be disclosed and in whether or not disclosures were limited to information relevant to the content/topic of the guideline.

**Table 4:** Disclosure of relationships of interest.

Organisation	What are the types of financial relationships considered? ^(a)^	Is financial threshold considered?	What are the types of nonfinancial relationships considered? ^(b)^	Is relevance to the guideline topic considered?	Is time period for disclosure considered?	About whom is DOI collected?	Is information for disclosure on financial and/or nonfinancial relationships of the individual’s personal relationships considered?
ACP [35]	Paid employment. Paid consultancy or speaking engagement, honoraria, advisory role, board membership. Research grant or salary support. Patent or royalties. Equity/stock or shares. Gift. Others	No	Development of related guidelines and standards, educational material. Having personal convictions (political, religious, ideological or other) related to the guideline topic that may interfere with an unbiased evidence review or recommendation process. Member of advisory board, committee, organisations, advocacy group. Others	Yes	Yes. Present and within the past three years	Members ACP, board of regents, clinical guideline committee and ACP staff	Yes. “Spouse” and limited to nonfinancial relationships “friend, spouse, family member, current or previous mentor or adversary”
CTFPHC [37]	Paid consultancy or speaking engagement, honoraria, advisory role, board membership. Research grant or salary support. Equity/stock or shares. Gift. Others	No	Publications, trials, systematic reviews. Member of advisory board, committee, organisations, advocacy group. Others	Yes	No	Potential participant in a CTFPHC led initiatives (peer reviewer, clinical expert, review team member, CTFPHC members)	Yes. “Spouse and immediate family members”
CDC-ACIP [39,40]	Paid employment. Paid consultancy or speaking engagement, honoraria, advisory role, board membership. Patent or royalties. Others	No	Not reported	No	No	Members of CDC-ACIP	Yes. “Immediate family member”
CENETEC [41]	Paid employment. Paid consultancy or speaking engagement, honoraria, advisory role, board membership. Patent or royalties	No	Others	Yes	No	Members of the guideline development group	No
FMSD [43]	Paid consultancy or speaking engagement, honoraria, advisory role, board membership. Research grant or salary support. Patent or royalties. Others	No	Not reported	No	Yes. 36 months prior submission of work only for some types of relationships	Members of the guideline development group	No
IETS [44]	Paid employment. Paid consultancy or speaking engagement, honoraria, advisory role, board membership. Research grant or salary support. Equity/stock or shares. Others	No	Development of related guidelines and standards, educational material. Publications, trials, systematic reviews. Others	No	Yes. 24 months before only for some types of relationships	Potential participants in the guideline work, any individual who has direct input to the guideline (members of the guideline development group: clinicians, patients, methodologists, external advisors, peer reviewers)	Yes. First-degree relative, spouse, partner (*pareja de hecho*), children for whom the member is legally responsible
AWMF [45]	Paid employment. Paid consultancy or speaking engagement, honoraria, advisory role, board membership. Research grant or salary support. Patent or royalties. Equity/stock or shares.	No	Having personal convictions (political, religious, ideological or other) related to the guideline topic that may interfere with an unbiased evidence review or recommendation process. Member of advisory board, committee, organisations, advocacy group	No	Yes. Within the last 3 years	Steering committee members, coordinators and work group leaders, and all participants in the guideline work	Yes. “Personal/professional partners”
GS [47]	Paid consultancy or speaking engagement, honoraria, advisory role, board membership. Research grant or salary support. Equity/stock or shares. Others	No	Not reported	No	Yes. Present and 3 years before	Participants in the guideline work and anyone who has direct input into the guideline (including experts, patient/caregiver)	No
HAS [48,49]	Paid employment. Paid consultancy or speaking engagement, honoraria, advisory role, board membership. Research grant or salary support. Patent or royalties. Equity/stock or shares. Others	No	Publications, trials, systematic reviews. Member of advisory board, committee, organisations, advocacy group. Others	No	Yes. Present and 5 years before	Members of the guideline development group, individual experts, personnel of HAS	Yes. Spouse, cohabitant, *pacsè* (who has signed the *pacte civil de solidarité*), parents and the children of this; individual’s children, parents
NCEC [55,56]	Paid employment. Paid consultancy or speaking engagement, honoraria, advisory role, board membership. Patent or royalties. Equity/stock or shares. Gift	No	Development of related guidelines and standards, educational material	No	No	Committee members of NCEC, members of the guideline development group	No
NHMRC [57]	Paid employment. Paid consultancy or speaking engagement, honoraria, advisory role, board membership. Research grant or salary support. Equity/stock or shares. Gift. Others	No	Development of related guidelines and standards, educational material. Publications, trials, systematic reviews. Member of advisory board, committee, organisations, advocacy group. Others	Yes	Yes. Over the past 3 years	Chair and other members of the guideline development group	Yes. “Immediate family members (partner and dependent children)”
NICE [58,59]	Paid employment. Paid consultancy or speaking engagement, honoraria, advisory role, board membership. Research grant or salary support. Equity/stock or shares. Patent or royalties. Others	No	Development of related guidelines and standards, educational material. Publications, trials, systematic reviews. Member of advisory board, committee, organisations, advocacy group. Others	No	Yes. 12 months before joining an advisory committee or during the period of membership of an advisory committee	All Committee members and anyone who has direct input into the guideline including the developer, the evidence review team, the expert witnesses	Yes. “Spouse or partner living in the same residence as the individual, as well as children and adults (who may or may not be living in the same residence) for whom the individual is legally responsible”
SIGN [61,62]	Paid employment. Paid consultancy or speaking engagement, honoraria, advisory role, board membership. Research grant or salary support. Equity/stock or shares. Gift. Others	No	Member of advisory board, committee, organisations, advocacy group	No	Yes. The year prior to the declaration, and the year following the declaration	Members of SIGN and anyone who has direct input to the guideline (members of the guideline development group, peer reviewers, advisors)	Yes. “Partners or close relatives”
SNLG [63]	Paid employment. Paid consultancy or speaking engagement, honoraria, advisory role, board membership. Equity/stock or shares. Patent or royalties	No	Not reported	Yes	No	Members of guideline development group	No
TGL [64]	Paid consultancy or speaking engagement, honoraria, advisory role, board membership. Research grant or salary support. Equity/stock or shares. Patent or royalties. Others	No	Member of advisory board, committee, organisations, advocacy group. Others	Yes-	Yes. Five past years, current and planned activities	Director and staff of TLG, members of expert group, external reviewers	Yes. “Associate: a member of the person’ family, or a business or professional colleague or partner”
GETS [65]	Paid employment. Paid consultancy or speaking engagement, honoraria, advisory role, board membership. Research grant or salary support. Equity/stock or shares. Gift. Others	No	Development of related guidelines and standards, educational material	No	Yes. Last 3 years	Any individual who has direct input to the guidelines including external collaborators and patients	Yes. Familiar
HE-TU [66]	Paid employment. Paid consultancy or speaking engagement, honoraria, advisory role, board membership. Research grant or salary support. Equity/stock or shares. Patent or royalties	Yes	Member of advisory board, committee, organisations, advocacy group. Others	Yes	Yes. Within the past 3 years	Panel members. The chair of the panel	Yes. “Spouse, adult children or siblings, close professional colleagues, administrative unit or department “
USPSTF [67]	Paid employment. Paid consultancy or speaking engagement, honoraria, advisory role, board membership. Equity/stock or shares. Patent or royalties	Yes	Member of advisory board, committee, organisations, advocacy group. Others	Yes	Yes. Two months prior to the meeting and continues until the final reports are completed. Past five years, in case of expert testimony or expert review in a medical malpractice case on a given Task Force-related topic	Task Force members	Yes. “Spouse and dependent children”
WHO [68]	Paid employment. Paid consultancy or speaking engagement, honoraria, advisory role, board membership. Research grant or salary support. Equity/stock or shares. Patent or royalties. Others	Yes	Publications, trials, systematic reviews. Member of advisory board, committee, organisations, advocacy group. Others	Yes	No	Members of the guideline development group, systematic review team, methodologists, external review group if they represent themselves	Yes. “Individual’s immediate family members (defined as the spouse, or partner with whom one has a close personal relationship, and the children)”

^**(a)**^ **Types of financial relationships considered to perform the content analysis** Paid employment. Paid consultancy or speaking engagement, honoraria, advisory role, board membership. Research grant or salary support. Patent or royalties. Equity/stock or shares. Gift. Others (e.g., travel grant, attending educational events)

^**(b)**^ **Type of nonfinancial relationships considered to perform the content analysis** Development of related guidelines and standards, educational material. Publications, trials, systematic reviews. Having personal convictions (political, religious, ideological or other) related to the guideline topic that may interfere with an unbiased evidence review or recommendation process. Member of advisory board, committee, organisations, advocacy group. Others (e.g., expert testimony, public statement, speech, lecture, opinion leader, other reputational risks)

Concerning the item “who is required to report on relationships of interest”, all policies stated that a declaration is collected from members of the guideline development group/panel/committee but a minority [37,44,58,59,61,62,68] (5/19, 26%) detailed additional disclosures from staff involved in a standard guideline development process, for example, guideline methodologists, systematic review team, reviewers and advisors. In contrast, policies were much more likely [35,37,39,40,44,45,48,49,57,58,59,61,62,64–68] (14/19, 74%) to require the disclosure of close personal relationships.

### Procedures for collecting, reviewing DOI and managing COI

The majority of policies explicitly reported that DOI is requested before appointment of the members of the guideline development group [37,39,40,44,47,48,49,57–59,64–68] (12/19, 63%), while the remaining seven policies [35,41,43,45,55,56,61–63] (7/19, 37%) did not report or were unclear as to whether this important practice was actually used (Table 5).

**Table 5:** Strategies for disclosure and management of conflict of interest and for recording and making disclosure accessible.

Organisation	Is it reported if disclosure of relationships of interest is required prior to selection of guideline development group members?	Is it reported who reviews disclosure of relationships of interests and makes decisions?	Is it reported if assessment of risk is performed?	Is it reported if divestment is required?	Is it reported if a procedure of exclusion is applied?	Is it reported if there are any relationships of interest specifically prohibited?	Are there reported penalties for those who fail to disclose interests?	Is there any description of the process to record disclosure of relationships of interest?	Is the completed original disclosure of relationships of interest form reported as published?
ACP [35]	No	Yes. At each meeting the Clinical Guideline Committee declares discusses and resolves conflict of interest of Clinical Guideline Committee-members and ACP staff	No	No	Yes	No	No	No	Not applicable
CTFPHC [37]	Yes	Yes. On appointment, the Joint Appointment Committee has the final decision. During membership the office of Public Health Agency of Canada reviews the disclosure form with chair of the CTFPHC topic group and CTFPHC chair or vice-chair	No	No	Yes	Yes, but limited to accepting more than $10.000 per year per expert testimony	No	Yes	Yes. Published in the website
CDC-ACIP [39,40]	Yes	Yes. On appointment, the Secretary of the US Department of Health and Human Services. Unclear during membership	No	Yes	Yes	Yes	No	No	No
CENETEC [41]	No	No	No	No	No	No	No	Yes	Unclear
FMSD [43]	Unclear	No	No	No	Yes	No	No	Yes	Unclear
IETS [44]	Yes	Yes. Members of the guideline development group, independent committee in case of disagreement	Yes	No	Yes	No	No	Yes	Yes. Published in the website and in the guideline
AWMF [45]	Unclear	Yes. Board of delegating medical societies appraise disclosure of the steering committees and coordinators. Steering committee and coordinators appraise the conflict of interest of the other participants in guideline development	No	No	Yes	No	No	Yes	Unclear
GS [47]	Yes	Yes. On appointment, the chair of the guideline development group and anyone who contribute significantly to coordination of the project	No	No	Yes	No	No	Yes	Yes. In the guideline
HAS [48,49]	Yes	Yes. On appointment the responsible officer prepares a table of the declaration of relationships of interest and presents a selection of the experts to the responsible board that confirms the group composition. Unclear during membership	Yes	No	Yes	No	Yes	Yes	Yes. In the website
NCEC [55,56]	Unclear	Yes. Chair of the NCEC is responsible for managing conflict of interest	No	No	Yes	No	No	Yes	No. The interest will be recorded on the committee’s register of interests, which will be maintained by the committee administrative support
NHRMC [57]	Yes	Yes. On appointment the chief executive officer or delegates. During membership the chair of the guideline group has the final decision	No	Yes	Yes	No	Yes	Yes	Yes. In the website before work commences and in the final guideline unless exception is granted by NHRMC chief executive officer
NICE [58,59]	Yes	Yes. During membership, for the committee members the chair. In case of disagreement between the chair and members of the committee the relevant NICE director. On appointment another policy on recruitment is referred to	Yes	No. Only NICE Board members	Yes	No	No	Yes	Unclear
SIGN [61,62]	Unclear	Yes. SIGN senior management team considers eligibility for guideline development group membership. Disclosure of relationships of interest monitored by chair of guideline development group during membership	No	No	Yes	No	No	Yes	No. Summary at SIGN website
SNLG [63]	No	No	No	No	No	No	No	No	Not applicable
TGL [64]	Yes	Yes. On appointment chief executive officer of TGL. During membership the chair of the guideline group has the final decision. Alternatively referred to the TGL Board	No	No	Yes	No	No	Yes	Yes. In the website
GETS [65]	Yes	Yes. Project leader and chair during membership. Unclear on appointment	No	No	Yes	No	No	No	Not applicable
HE-TU [66]	Yes	Yes. On appointment the Guideline advisory board. Chair during membership	No	No	Yes	No	No	Yes	Yes. In the guideline
USPSTF [67]	Yes	Yes. A committee composed of AHRQ staff and the USPSTF chair and vice chair review each member's disclosures and issue a recommendation on new USPSTF members and on the member's eligibility to participate in a specific guideline	No	No	Yes	Yes. But limited to accept more than $ 10.000 per year per expert testimony or expert review for medical malpractice cases	No	Yes	No. Kept on file at AHRQ. It is possible that information may be shared with the public if requested under the Freedom of Information Act
WHO [68]	Yes	Yes. Disclosure of members of the guideline development group reviewed by the responsible technical officer and director, with input from office of compliance, risk management and ethics (CRE) as needed. Disclosure of methodologist, systematic review team, external review group by steering group with input from CRE as needed	Yes	No	Yes	No	No	Yes	No. Summary in the guideline

Regarding the item “who reviews DOI and makes decision” of the 16 organisations reporting on this issue [35,37,39,40,44,45,47–49,55–59,61,62,64–68] (16/19, 84%) the chair and/or the members of the guideline development group were the most cited responsible entity [35,37,44,45,47,57–59,61,62,64–66] (11/16, 69%). In contrast, only 2 organisations [67,68] (2/16, 13%) appeared to rely exclusively on a committee as an independent entity to review DOI and to devise a management plan across the entire guideline development process.

All organisations reported procedures for exclusion of members with conflicts but divestment was considered a requirement only by 2 organisations [39,40,57] (2/19, 11%), and prohibition of specific relationships by 3 organisations [37,39,40,67]. Only 4 policies [44,48,49,58,59,68] (4/19, 21%) specified an “assessment of risk”, defined as a procedure and criteria to assess whether a relationship of interest constitutes a COI and to evaluate the potential harm of the COI identified [13].

### Procedures for recording and public disclosure of DOI

The majority of organisations reported a description of the procedures to record DOI [37,41,43–45,47–49,55–59,61,62,64,66–68] (15/19, 79%) whereas four did not address this issue [35,39,40,63,65] (4/19, 21%) (Table 5).

Of those policies that described activities to record DOI, the original completed DOI forms were reported to be publicly accessible on the website and/or in the published guideline by seven organisations [37,44,47,48,49,57,64,66] (7/15, 47%) with some exceptions. For example, one organisation stated DOI will be made public on the organisation website but”exception to this requirement will be by Chief Executive Officer in appropriate circumstances” [57].

Two organisations published in the guideline and/or website a summary of the DOI information [61,62,68] while other two [55,56,67] kept this information confidential although one of these mentioned the possibility of access upon request under state law [67].

## Discussion

We found that of the 29 organisations corresponding to our eligibility criteria we were not able to identify COI policies for approximately one in four organisations (8/29, 28%). Failure of guideline developer organisations to adopt a publicly accessible COI policies related to guideline development has also been reported elsewhere in literature. For example, in the above mentioned cross-sectional study of Norris et al. [30] only 46% of the 37 surveyed organisations had a COI policy directly related to health care guidelines. These findings along with the data from our study suggest that the absence of a COI policy might be one of the reason behind the phenomenon of underreporting of COI in guidelines [23,26,27,29] as well as the observed uneven adherence to current standards on COI in guidelines [18–21].

We also found that many organisations provided no or unclear information pertaining to some items of our data abstraction instrument. In our view, a particularly troubling area in which COI policies need to be improved is that of guideline panel selection and composition. More attention to this issue has been advocated to reduce of the potential for COI to create bias [10,12,28]. Nevertheless, in our investigation about one-third of the policies did not report or did not clearly report whether disclosure was a prerequisite for panel selection. Additionally, preventive strategy such as divestment [13,15] and prohibition [13] were rarely mentioned.

A second key area in which COI policies need to be improved is the management of the disclosed information, particularly regarding the practices of “the assessment of risk” which is defined as “practices and criteria used to determine if a relation of interest constitutes a COI and to assess the potential for harm of the COI identified” [13]. It is encouraging that the policies surveyed were directed at the most common relationships of interest in support of the identification of COI. However a policy for systematic and transparent management of the disclosed information was uncommon. Few organisations clearly stated what practices and criteria were used to determine whether a relation of interest constitutes a COI and to assess the potential for harm of the COI identified. The translation of the disclosed information into a decision or measures taken to limit the likelihood of undue influence of COI is a critical step. This step should be explicit and transparent to reduce the flexibility of the organisations in addressing financial and nonfinancial ties as well as to assure the ability of the informed reader to appraise the validity of decisions on COI issue.

In terms of the management and monitoring of the disclosed information IOM recommends that organisations should create a COI committee (Recommendation 3.1) [13]. In this regard we found that few organisations have felt the need or identified the necessary resources to have a dedicated infrastructure to act as a third party providing independent review of DOI and managing COI across all the phases of guideline development process.

Most organisations relied on the same group of individuals who develop the guidelines to handle the disclosed cases. This is of particular concern if considered in the context of the evidence of guideline chairs and panel members with a high proportion of conflicts [20,25]. Apart from an improved consistency in decisions taken across guidelines, in our view a “conflict of interest committee” could avoid administrative burden for the chair, co-chair and members of the guideline group and more importantly could consistently implement procedures for the monitoring and enforcement of the policies.

Furthermore our study underlines the issue of variation across policies in keeping with the findings of Norris et al. [30]. We found that the policies varied in their content, for example in the definition of COI (when provided), in the categories and details of the information required to be disclosed, in the time frame for disclosure, in establishing or not establishing a threshold, and in detailing who is required to declare a relationship of interest. Our sample included organisations from 13 countries and one intergovernmental organisation, so this variation in part probably reflects cultural, legal and administrative differences across countries as well as differences in organisational values and interests.

We noted that the policies were also variable in format and accessibility across the organisations, limiting the ability of the guideline user to obtain a clear and complete picture of the strategy to address COI throughout the guideline development process. An important finding is that less than half of the included organisations reported that the information from completed DOI forms was publicly accessible without restriction. Again, notwithstanding cultural, legal and administrative issues, the guideline user has the right to assess the completeness of the disclosure of COI as well as the consistency with which the policy is implemented.

This study contributes to the literature on the quality of guidelines by demonstrating, using an innovative sampling method and highly detailed data extraction, that COI remains an area that requires attention from guideline users and improvement from many guideline development organisations. The study confirms previous research that has drawn the same conclusions about COI from different samples of guidelines. The study also has some limitations that deserve comment.

First, organisations may have practices which are not fully reflected in the written COI policies or do have policy but not publicly available. However, we decided to rely only on information on COI policies publicly accessible on website since we consider accessibility an essential element of a transparent COI policy. As pointed out by IOM, COI policies should be comprehensible and accessible to the individuals and institutions that may be affected [13]. We recommend that the organisations that disseminate their own health care guidelines should provide public information on their strategies to identify and manage COI through an understandable and accessible policy.

Second, information about the policies and information about COI within the policies might be incomplete. We experienced several difficulties in locating COI policies from websites and in obtaining a complete and clear picture of the actual information required to be disclosed, the process of reviewing and the management of COI for the entire guideline development process. Some organisations published their COI policies as a complete self-contained document on their website, whereas others included information on identification and management of COI in their guideline methodology document. In some instances information was interspersed among diverse parts of the guideline methodological document, the disclosure of interest form and other supporting policies. Given the unstructured nature of the documents retrieved we checked as far as possible for presence of information about COI. We also encountered problems in the interpretation and coding of the information (e.g., on what should be disclosed) given that there is not an agreed taxonomy of terms relating to COI.

Third, we restricted our study to organisations that produce guidelines on a wide range of health topics and we excluded some professional organisations, which may limit the generalizability of our results. However, we would argue that our inclusion criteria would bias the sample towards organisations with greater resources for guideline development and therefore, if anything, our results would underestimate the extent of the problem of inadequate COI management. However our study has the strength of including 29 organisations from 19 different countries and one intergovernmental organisation in the sample and a content analysis of documents in four different languages. In terms of countries and languages represented, this may be a more representative sample of guideline development organisations than those obtained in other studies of COI in guidelines.

## Conclusions

The finding that some organisations despite the recommendations and standards issued respectively in 2009 [13], 2011 [14] and 2012 [15] still do not have COI policies publicly available is concerning. Also troubling was the failure of many policies to clearly report on critical steps of obtaining, managing and communicating disclosure of relationships of interest. These problems in addition to the existing variation among policies may increase the scope for inconsistency in addressing COI issues; furthermore, unclear and incomplete COI policies may confuse readers, erode public confidence, and decrease trust in guidelines and the organisations that produce them. Thus, it is in the interest of guideline users and developers to promote strong adherence to the standards available on COI in conjunction with better scientific journal policies so that every guideline is completed by a harmonized, complete, understandable, and accessible COI policy.

## Supporting Information

S1 Table Search strategy for evidence inventory of surveys of guideline developer organisations. (DOCX)

S2 Table List of 92 guideline developer organisations. (DOCX)

## References

[1] Johnson L, Stricker RB. Attorney General forces Infectious Diseases Society of America to redo Lyme guidelines due to flawed development process. J Med Ethics. 2009 5;35(5):283–8. 10.1136/jme.2008.026526 19407031

[2] Lenzer J. French guidelines are withdrawn after court finds potential bias among authors. BMJ. 2011 6 24;342:d4007 10.1136/bmj.d4007 21705408

[3] McSweegan E. Lyme disease and the politics of public advocacy. Clin Infect Dis. 2008 12 15;47(12):1609–10. 10.1086/595684 19025374

[4] Shaneyfelt TM, Centor RM. Reassessment of clinical practice guidelines: go gently into that good night. JAMA. 2009 2 25;301(8):868–9. 10.1001/jama.2009.225 19244197

[5] Sniderman AD, Furberg CD. Why guideline-making requires reform. JAMA. 2009 1 28;301(4):429–31. 10.1001/jama.2009.15 19176446

[6] Akl EA, El-Hachem P, Abou-Haidar H, Neumann I, Schünemann HJ, Guyatt GH. Considering intellectual, in addition to financial, conflicts of interest proved important in a clinical practice guideline: a descriptive study. J Clin Epidemiol. 2014 11;67(11):1222–8. 10.1016/j.jclinepi.2014.05.006 24970099

[7] Boyd EA, Bero LA. Improving the use of research evidence in guideline development: 4. Managing conflicts of interests. Health Res Policy Syst. 2006 12 1;4:16 10.1186/1478-4505-4-16 17140441 PMC1693552

[8] Guyatt G, Akl EA, Hirsh J, Kearon C, Crowther M, Gutterman D, et al The vexing problem of guidelines and conflict of interest: a potential solution. Ann Intern Med. 2010 6 1;152(11):738–41. 10.7326/0003-4819-152-11-201006010-00254 20479011

[9] Rothman DJ, McDonald WJ, Berkowitz CD, Chimonas SC, De Angelis CD, et al Professional medical associations and their relationships with industry: a proposal for controlling conflict of interest. JAMA. 2009 4 1;301(13):1367–72. 10.1001/jama.2009.407 19336712

[10] Lenzer J, Hoffman JR, Furberg CD, Ioannidis JP, Guideline Panel Review Working Group. Ensuring the integrity of clinical practice guidelines: a tool for protecting patients. BMJ. 2013 9 17;347:f5535 10.1136/bmj.f5535 24046286

[11] Schünemann HJ, Osborne M, Moss J, Manthous C, Wagner G, Sicilian L, et al An official American Thoracic Society Policy statement: managing conflict of interest in professional societies. Am J Respir Crit Care Med. 2009 9 15; 180(6): 564–80. 10.1164/rccm.200901-0126ST 19734351

[12] Sniderman AD, Furberg CD. Pluralism of viewpoints as the antidote to intellectual conflict of interest in guidelines. J Clin Epidemiol. 2012 7;65(7):705–7. 10.1016/j.jclinepi.2012.01.009 22542359

[13] Institute of Medicine (IOM). Conflict of Interest in Medical Research, Education, and Practice. Washington, D.C: The National Academies Press; 2009. 20662118

[14] Institute of Medicine (IOM). Clinical Practice Guidelines We Can Trust. Washington, DC: The National Academies Press; 2011.

[15] Qaseem A, Forland F, Macbeth F, Ollenschläger G, Phillips S, van der Wees P, et al Guidelines International Network: toward international standards for clinical practice guidelines. Ann Intern Med. 2012 4 3;156(7):525–31. 10.7326/0003-4819-156-7-201204030-00009 22473437

[16] Schünemann HJ, Al-Ansary LA, Forland F, Kersten S, Komulainen J, Kopp IB, et al Guidelines International Network: Principles for Disclosure of Interests and Management of Conflicts in Guidelines. Ann Intern Med. 2015 10 6;163(7):548–53. 10.7326/M14-1885 26436619

[17] Brouwers MC, Kho ME, Browman GP, Burgers JS, Cluzeau F, Feder G, et al AGREE II: advancing guideline development, reporting and evaluation in health care. CMAJ. 2010 12 14;182(18):E839–42. 10.1503/cmaj.090449 20603348 PMC3001530

[18] Holmer HK, Norris SL, Ogden LA, Burda BU, Fu R. Conflicts of interest among authors of clinical practice guidelines for glycemic control in type 2 diabetes mellitus. PLoS One. 2013 10 14;8(10):e75284 10.1371/journal.pone.0075284 24155870 PMC3796568

[19] Feuerstein JD, Akbari M, Gifford AE, Cullen G, Leffler DA, Sheth SG, et al Systematic review: the quality of the scientific evidence and conflicts of interest in international inflammatory bowel disease practice guidelines. Aliment Pharmacol Ther. 2013 5;37(10):937–46. 10.1111/apt.12290 23550536

[20] Kung J, Miller RR, Mackowiak PA. Failure of clinical practice guidelines to meet institute of medicine standards: Two more decades of little, if any, progress. Arch Intern Med. 2012 11 26;172(21):1628–33. 10.1001/2013.jamainternmed.56 23089902

[21] Knai C, Brusamento S, Legido-Quigley H, Saliba V, Panteli D, Turk E, et al Systematic review of the methodological quality of clinical guideline development for the management of chronic disease in Europe. Health Policy. 2012 10;107(2–3):157–67. 10.1016/j.healthpol.2012.06.004 22795610

[22] Choudhry NK, Stelfox HT, Detsky AS. Relationships between authors of clinical practice guidelines and the pharmaceutical industry. JAMA. 2002 2 6;287(5):612–7. 11829700 10.1001/jama.287.5.612

[23] Bindslev JB, Schroll J, Gøtzsche PC, Lundh A. Underreporting of conflicts of interest in clinical practice guidelines: cross sectional study. BMC Med Ethics. 2013 5 3;14:19 10.1186/1472-6939-14-19 23642105 PMC3651727

[24] Mendelson TB, Meltzer M, Campbell EG, Caplan AL, Kirkpatrick JN. Conflicts of interest in cardiovascular clinical practice guidelines. Arch Intern Med. 2001 3 28; 171: 577–84. 10.1001/archinternmed.2011.96 21444849

[25] Moynihan RN, Cooke GP, Doust JA, Bero L, Hill S, Glasziou PP. Expanding disease definitions in guidelines and expert panel ties to industry: a cross-sectional study of common conditions in the United States. PLoS Med. 2013 8;10(8):e1001500 10.1371/journal.pmed.1001500 23966841 PMC3742441

[26] Neuman J, Korenstein D, Ross JS, Keyhani S. Prevalence of financial conflicts of interest among panel members producing clinical practice guidelines in Canada and United States: cross sectional study. BMJ. 2011 10 11; 343:d5621 10.1136/bmj.d5621 21990257 PMC3191201

[27] Norris SL, Holmer HK, Ogden LA, Burda BU. Conflict of interest in clinical practice guideline development: a systematic review. PLoS One. 2011;6(10):e25153 10.1371/journal.pone.0025153 22039406 PMC3198464

[28] Norris SL, Burda BU, Holmer HK, Ogden LA, Fu R, Bero L, et al Author's specialty and conflicts of interest contribute to conflicting guidelines for screening mammography. J Clin Epidemiol. 2012 7;65(7):725–33. 10.1016/j.jclinepi.2011.12.011 22498428

[29] Norris SL, Holmer HK, Ogden LA, Selph SS, Fu R. Conflict of interest disclosures for clinical practice guidelines in the national guideline clearinghouse. PLoS One. 2012;7(11):e47343 10.1371/journal.pone.0047343 23144816 PMC3492392

[30] Norris SL, Holmer HK, Burda BU, Ogden LA, Fu R. Conflict of interest policies for organisations producing a large number of clinical practice guidelines. PLoS One. 2012;7(5):e37413 10.1371/journal.pone.0037413 22629391 PMC3358298

[31] Hartling L, Guise J-M, Kato E, Anderson J, Aronson N, Belinson S, et al. EPC Methods: An Exploration of Methods and Context for the Production of Rapid Reviews. Research White Paper. (Prepared by the Scientific Resource Center under Contract No. 290-2012-00004-C.) AHRQ Publication No. 15-EHC008-EF. Rockville, MD: Agency for Healthcare Research and Quality; 2015. 25654160

[32] Legido-Quigley H, Panteli D, Brusamento S, Knai C, Saliba V, Turk E, et al Clinical guidelines in the European Union: mapping the regulatory basis, development, quality control, implementation and evaluation across member states. Health Policy. 2012 10;107(2–3):146–56. 10.1016/j.healthpol.2012.08.004 22939646

[33] Legido-Quigley H, Panteli D, Car J, McKee M, Busse R, editors. Clinical guidelines for chronic condition in the European Union European observatory on health system and policies. Geneva, World Health Organization; 2013 (Observatory Studies Series 30).

[34] Schünemann HJ, Wiercioch W, Etxeandia I, Falavigna M, Santesso N, Mustafa R, et al Guidelines 2.0: systematic development of a comprehensive checklist for a successful guideline enterprise. CMAJ. 2014 2 18;186(3):E123–42. 10.1503/cmaj.131237 24344144 PMC3928232

[35] Qaseem A, Snow V, Owens DK, Shekelle P, Clinical Guidelines Committee of the American College of Physicians. The development of clinical practice guidelines and guidance statements of the American College of Physicians: summary of methods. Ann Intern Med. 2010 8 3; 153(3):194–9. 10.7326/0003-4819-153-3-201008030-00010 20679562

[36] Federaal Kenniscentrum voor de Gezondheidszorg (Belgian Health Care Knowledge Centre). Process book. Available: http://processbook.kce.fgov.be/ Last access: 15 June 2015.

[37] Canadian Task Force on Preventive Health Care. Procedure Manual; March 2014. Available: http://canadiantaskforce.ca/files/procedural-manual-en.pdf Last access: 15 June 2015.

[38] Umscheid CA, Agarwal RK, Brennan PJ, for the Healthcare Infection Control Practices Advisory Committee (HICPAC). Updating the Guideline Methodology of the Healthcare Infection Control Practices Advisory Committee (HICPAC). Available: http://www.cdc.gov/hicpac/guidelineMethod/guidelineMethod.html Last access: 17 June 2015. 10.1016/j.ajic.2009.12.005 20116133

[39] Ahmed F. U.S. Advisory Committee on Immunization Practices Handbook for Developing Evidence-based Recommendations. Version 1.2. Atlanta, GA: Centers for Disease Control and Prevention (CDC); 2013 Available: http://www.cdc.gov/vaccines/acip/recs/grade/downloads/handbook.pdf Last access: 17 June 2015.

[40] Smith JC, Snider DE, Pickering LK, Advisory Committee on Immunization Practices. Immunization policy development in the United States: the role of the Advisory Committee on Immunization Practices. Ann Intern Med. 2009 1 6;150(1):45–9. Available: http://www.cdc.gov/vaccines/acip/committee/index.html Last access: 17 June 2015. 19124820 10.7326/0003-4819-150-1-200901060-00009

[41] Centro National de Excelentia Tecnólogica en Salud. Metodología para la Integración de Guías de Práctica Clínica; 2007. Available: http://www.cenetec.salud.gob.mx/descargas/gpc/METODOLOGIA_GPC.pdf Last accessed: 17 June 2015.

[42] Conseil Scientifique du Domaine de la Santé. Procédures pour l’établissement et la diffusion de référentiels de bonne pratique par le Conseil Scientifique. Available: http://www.conseil-scientifique.lu/index.php?id=27 Last access: 17 June 2015.

[43] Finnish Medical Society Duodecim Current Care Guidelines. Disclosure of COI; 2014. Available: http://www.terveysportti.fi/xmedia/ccs/process/Sidonnaisuudet.html Last access: 17 June 2015.

[44] Instituto de Evaluación Tecnológica en Salud. Guía Metodológica para la elaboración de Guías de Práctica Clínica con Evaluación Económica en el Sistema General de Seguridad Social en Salud; 2014. Available: http://www.iets.org.co/Manuales/Manuales/Gu%C3%ADa%20Metodol%C3%B3gica%20Elaboraci%C3%B3n%20de%20GPC%20con%20Evaluaci%C3%B3n%20Econ%C3%B3mica%20en%20el%20Sist%20Seguridad%20Social%20y%20Salud-Versi%C3%B3n%20final%20completa.pdf Lat access: 17 June 2015.

[45] German Association of the Scientific Medical Societies (AWMF), Standing Guidelines Commission. AWMF Guidance Manual and Rules for Guideline Development, 1st Edition 2012. English version. Available: http://www.awmf.org/fileadmin/user_upload/Leitlinien/AWMF-Regelwerk/AWMF-Guidance_2013.pdf Last access: 17 June 2015.

[46] German Medical Association (GMA), National Association of Statutory Health Insurance Physicians (NASHIP), Association of the Scientific Medical Societies (AWMF). National Program for Disease Management Guidelines. Method Report. 4th edition. 2010. Available: http://www.leitlinien.de/mdb/downloads/nvl/methodik/mr-engl-aufl-4-version-1.pdf Last access: 17 June 2015.

[47] Grupo de trabajo sobre GPC Elaboración de Guías de Práctica Clínica en el Sistema Nacional de Salud. Manual Metodológico. Madrid: Plan Nacional para el SNS del MSC. Instituto Aragonés de Ciencias de la Salud-I+CS; (2007). Available: http://www.guiasalud.es/emanuales/elaboracion/documentos/Manual%20metodologico%20-%20Elaboracion%20GPC%20en%20el%20SNS.pdf Last access: 17 June 2015.

[48] Haute Autorité de Santé. Guide des déclarations d’intérêts et de gestion des conflits d’intérêts. Guide de prévention et de gestion des conflits d’intérêts adopté par le Collège le 24 juillet 2013. Available: http://www.has-sante.fr/portail/upload/docs/application/pdf/guide_dpi.pdf Last access: 17 June 2015.

[49] Haute Autorité de Santé. Guide Mèthodologique. Élaboration de recommandations de bonne pratique; 2010. Mise à jour: Mars 2015. Available: http://www.has-sante.fr/portail/upload/docs/application/pdf/2011-01/guide_methodologique_recommandations_pour_la_pratique_clinique.pdf Last access: 17 June 2015.

[50] Davino-Ramaya C, Krause LK, Robbins CW, Harris JS, Koster M, Chan W, et al Transparency matters: Kaiser Permanente's National Guideline Program methodological processes. Perm J. 2012 Winter;16(1):55–62. Available: http://www.kpihp.org/wp-content/uploads/2013/07/KPStories-v2n2-ClinicalGuidelines-FINAL.pdf Last access: 17 June 2015. 22529761 10.7812/tpp/11-134 PMC3327114

[51] Ministerio de Salud. Norma técnica para la elaboratión de Guías de Práctica Clínica; 2006. Available: http://bvs.minsa.gob.pe/local/MINSA/1176_DGSP196.pdf Last access: 17 June 2015.

[52] Ministerio de Salud y Protección Social. Guía Metodológica para la elaboración de Guías de Atención Integral en el Sistema General de Seguridad Social en Salud colombiano; 2010. Available: www.minsalud.gov.co/Documentos%20y%20Publicaciones/GUIA%20METODOLOGICA%20PARA%20LA%20ELABORACI%C3%93N%20DE%20GU%C3%8DAS%20DE%20ATENCI%C3%93N%20INTEGRAL.pdf Last access: 17 June 2015.

[53] Socialstyrelsen (National Board of Health and Welfare). Guideline process. Available: http://www.socialstyrelsen.se/nationalguidelines/howwedrawuptheguidelines Last access: 17 June 2015.

[54] King Saud bin Abdulaziz University for Health Sciences, National and Gulf Center for Evidence Based Health Practice. Guidance for Clinical Practice Guideline Development, Adaptation and Endorsement; 2008. Available:http://ngcebm.ksau-hs.edu.sa/images/content/NGCEBHC-Draft-3-CPG-Guidance.pdf Last access: 17 June 2015.

[55] National Clinical Effectiveness Committee. Guideline developers manual; 2013. Available: http://health.gov.ie/wpcontent/uploads/2015/01/ncec_guideline_development_manual_january13.pdf Last access: 17 June 2015.

[56] National Clinical Effectiveness Committee Policy on transparency and management of conflicts of interest; 2012. Available: http://health.gov.ie/patient-safety/ Last access: 17 June 2015.

[57] National Health and Medical Research Council. Guideline Development and Conflicts of Interest: Identifying and Managing Conflicts of Interest of Prospective Members and Members of NHMRC Committees and Working Groups Developing Guidelines; 2012. Available: http://www.nhmrc.gov.au/_files_nhmrc/file/guidelines/developers/nh155_coi_policy_120710.pdf Last access: 17 June 2015.

[58] National Institute for Health and Care Excellence. Developing NICE guidelines: the manual; 2015. Available: http://www.nice.org.uk/article/pmg20/resources/non-guidance-developing-nice-guidelines-the-manual-pdf Last access: 17 June 2015. 26677490

[59] National Institute for Health and Care Excellence. Code of practice for declaring and dealing with conflicts of interest; 2014. Available: https://www.nice.org.uk/Media/Default/About/Who-we-are/Policies-and-procedures/Code-of-practice-for-declaring-and-managing-conflicts-of-interest.pdf Last access: 17 June 2015.

[60] National Institute of Quality and Innovations (Národný Inštitút Kvality a Inovácií). A guideline developers’ handbook; 2005. Available: http://www.quality.healthnet.sk/EBM/Guidelines_development_handbook_Slovak_version_v1.pdf Last access: 17 June 2015.

[61] Scottish Intercollegiate Guidelines Network. Policy on Declaration of Competing Interests. Available: http://www.sign.ac.uk/pdf/doi-policy.pdf Last access: 17 June 2015.

[62] Scottish Intercollegiate Guidelines Network. SIGN 50—A guideline developer’s handbook; 2014. Available: http://www.sign.ac.uk/pdf/sign50.pdf Last access: 17 June 2015.

[63] Sistema Nazionale Linee Guida. Il Programma nazionale per le linee guida. Manuale metodologico. Come produrre, diffondere e aggiornare raccomandazioni per la pratica clinica; maggio 2004. Available: http://www.snlg-iss.it/cms/files/Manuale_PNLG_0.pdf Last access: 17 June 2015.

[64] Therapeutic Guidelines. Limited conflict of interest policy; 2014. Available: http://www.tg.org.au/uploads/PDFs/ConflictOfInterestPolicy_19Feb2014.pdf Last access: 17 June 2015.

[65] Universidad Nacional de Colombia, Instituto de Investigaciones clinicas, Facultad de Medicina. Guía Metodológica para la elaboración de Guías de Práctica Clínica con Evaluación Económica en el Sistema General de Seguridad Social en Salud Colombiano. Available: http://www.gets.unal.edu.co/manual_gpc.html Last access: 17 June 2015.

[66] Estonian Health Insurance Fund, University of Tartu Ministry of Social Affair, WHO. Estonian handbook for guidelines development; 2011. Available: http://www.ut.ee/en and http://whqlibdoc.who.int/publications/2011/9789241502429_eng.pdf Last access: 17 June 2015.

[67] U.S. Preventive Services Task Force. Procedure manual; March 2014 (AHRQ publication NO.08-05118-EF). Available: http://www.uspreventiveservicestaskforce.org/Page/Name/methods-and-processes Last access: 17 June 2015.

[68] World Health Organization. Handbook for guideline development; 2014. Available: http://www.who.int/kms/handbook_2nd_ed.pdf Last access: 17 June 2015.

[69] Thompson DF. Understanding financial conflicts of interest. N Engl J Med. 1993 8 18;329(8):573–6. 10.1056/NEJM199308193290812 8336759

[70] Detsky AS. Sources of bias for authors of clinical practice guidelines. CMAJ. 2006 10 24;175(9):1033, 1035. 10.1503/cmaj.061181 17060643 PMC1609150

