# Supplementary material for: Policies on Conflicts of Interest in Health Care Guideline Development: A Cross-Sectional Analysis
Source: PLoS One. 2016 Nov 15;11(11):e0166485. doi: 10.1371/journal.pone.0166485 (PMC5113001; doi:10.1371/journal.pone.0166485)
Supplement: S1 Table — (DOCX) [file pone.0166485.s001.docx]

| **S1 Table Search strategy for evidence inventory of surveys of guideline developer organisations** |
| --- |
| ("practice guideline developer" OR "practice guideline development")  OR  "practice guidelines as topic"[MeSH Major Topic]  AND (("quality control"[MeSH Terms] OR ("quality"[All Fields] AND "control"[All Fields]) OR "quality control"[All Fields]) OR ("checklist"[MeSH Terms] OR "checklist"[All Fields]))  AND ("evidence-based medicine"[MeSH Terms] OR ("evidence-based"[All Fields] AND "medicine"[All Fields]) OR "evidence-based medicine"[All Fields] OR ("evidence"[All Fields] AND "based"[All Fields] AND "medicine"[All Fields]) OR "evidence based medicine"[All Fields]) |
