# Supplementary material for: Policies on Conflicts of Interest in Health Care Guideline Development: A Cross-Sectional Analysis
Source: PLoS One. 2016 Nov 15;11(11):e0166485. doi: 10.1371/journal.pone.0166485 (PMC5113001; doi:10.1371/journal.pone.0166485)
Supplement: S2 Table — (DOCX) [file pone.0166485.s002.docx]

| **S 2 Table. List of 92 guideline developer organisations** | | | | | | | |
| --- | --- | --- | --- | --- | --- | --- | --- |
| **N.** | **Country** | **Organisation** | **Acronym** | Web**site** | **Access date** | **Reasons**  **for inclusion/exclusion** | **Documents link/comments** |
| 1 | Argentina | Academia Nacional  de Medicina | ANM | <http://www.acamedbai.org.ar/> | 9 April 2015 | Excluded since it is not a guideline developer | http://publicaciones.ops.org.ar/publicaciones/otras%20pub/GuiadeGuias.pdf  It provides adaptation manual |
| 2 | Australia | National Health and Medical Research Council | NHMRC | http://www.nhmrc.gov.au/ | 17 June 2015 | Included. Full text of document assessed for eligibility | http://www.nhmrc.gov.au/_files_nhmrc/file/guidelines/developers/nh155_coi_policy_120710.pdf |
| 3 | Australia | The Joanna Briggs Institute | JBI | http://joannabriggs.org | 11 June 2015 | Excluded since it is not a guideline developer |  |
| 4 | Australia | Cancer Australia | CA | http://canceraustralia.gov.au/ |  | Excluded since it is on specific health topics/specialties |  |
| 5 | Australia | Therapeutic Guidelines Limited | TGL | http://www.tg.org.au/ | 17 June 2015 | Included. Full text of document assessed for eligibility | http://www.tg.org.au/uploads/PDFs/ConflictOfInterestPolicy_19Feb2014.pdf |
| 6 | Australia | Diagnostic Imaging Pathways | DIP | http://www.imagingpathways.health.wa.gov.au/ |  | Excluded since it is on specific health topics/specialties |  |
| 7 | Austria | Gesundheit Österreich | GOEG | http://www.goeg.at/ | 23 March 2015 | Excluded since it does not provide publicly accessible document on guidelines development or COI issue guideline related | Small part website in English |
| 8 | Belgium | Belgian Health Care Knowledge Centre/Federal Centre of Health Care Expertise | KCE | http://kce.fgov.be | 26 March 2015 | Included. Full text of document assessed for eligibility | It provides a process book at http://processbook.kce.fgov.be/ |
| 9 | Belgium | Belgian Centre for Evidence-Based Medicine | CEBAM | http://www.cebam.be/ | 10 June 2015 | Excluded since it does not provide publicly accessible document on guidelines development or COI issue guideline related | Page on description of what they do for recommendations  https://www.cebam.be/fr/Richtlijnen/Pages/default.aspx  Generic advice  https://www.cebam.be/fr/Richtlijnen/Pages/Hoe-een-richtlijn-maken.aspx |
| 10 | Belgium | Belgian Antibiotic Policy Coordination Committee | BAPCOC | http://www.bapcoc.be/ |  | Excluded since it is on specific health topics/specialties |  |
| 11 | Belgium | Domus Medica | DM | http://www.domusmedica.be/ | 9 April 2015 | Excluded since it does not provide documents in English, French, Italian, Spanish |  |
| 12 | Belgium | EBM PracticeNet | EBMPN | https://wwwacc.ebmpracticenet.be/ | 10 June2015 | Excluded since it does not provide publicly accessible document on guidelines development or COI issue guideline related |  |
| 13 | Belgium | European Region of the World Confederation for Physical Therapy | WCPT | http://www.erwcpt.eu/ |  | Excluded since it is on specific health topics/specialties |  |
| 14 | Brazil | [Associação Médica Brasileira](http://amb.org.br/) | AMB | http://amb.org.br/ | 9 April 2015 | Excluded since it does not provide documents in English, French, Italian, Spanish |  |
| 15 | Canada | Registered Nurses' Association of Ontario | RNAO | http://rnao.ca/ |  | Excluded since it is on specific health topics/specialties |  |
| 16 | Canada | Canadian Task Force on Preventive Health Care | CTFPHC | http://canadiantaskforce.ca/ | 15 June 2015 | Included. Full text of document assessed for eligibility | http://canadiantaskforce.ca/files/procedural-manual-en.pdf |
| 17 | Canada | Cancer Care Ontario | CCO | http://www.cancercare.on.ca/ |  | Excluded since it is on specific health topics/specialties |  |
| 18 | Collaborative organisation | Grading of Recommendations Assessment, Development and Evaluation Working Group | GRADE | http://www.gradeworkinggroup.org/ | 9 April 2015 | Excluded since it is not a guideline developer |  |
| 19 | Colombia | Ministerio de Salud y Protección Social | MSPS | http://www.minsalud.gov.co | 17 June 2015 | Included.Full text of document assessed for eligibility | www.minsalud.gov.co/Documentos%20y%20Publicaciones/GUIA%20METODOLOGICA%20PARA%20LA%20ELABORACI%C3%93N%20DE%20GU%C3%8DAS%20DE%20ATENCI%C3%93N%20INTEGRAL.pdf |
| 20 | Colombia | Instituto de Evaluación Tecnológica en Salud | IETS | <http://www.iets.org.co> | 17 June 2015 | Included. Full text of document assessed for eligibility | http://www.iets.org.co/Manuales/Manuales/Gu%C3%ADa%20Metodol%C3%B3gica%20Elaboraci%C3%B3n%20de%20GPC%20con%20Evaluaci%C3%B3n%20Econ%C3%B3mica%20en%20el%20Sist%20Seguridad%20Social%20y%20Salud-Versi%C3%B3n%20final%20completa.pdf |
| 21 | Colombia | Instituto National de Cancerología | INC | http://www.cancer.gov.co/ |  | Excluded since it is on specific health topics/specialties |  |
| 22 | Colombia | Universidad Nacional de Colombia,Instituto de Investigaciones clinicas, Facultad de Medicina | GETS | http://www.gets.unal.edu.co/ | 17 June 2015 | Included. Full text of document assessed for eligibility | http://www.gets.unal.edu.co/manual_gpc.html |
| 23 | Czech Republic | [Verlag Dashöfer](http://www.dashofer.cz/) | VD | http://www.dashofer.cz/ | 9 April 2015 | Excluded since it does not provide documents in English, French, Italian, Spanish |  |
| 24 | Czech Republic | Národní Referenční Centrum | NRC | <http://www.nrc.cz/en> | 9 April 2015 | Excluded since it does not provide publicly accessible document on guidelines development or COI issue guideline related |  |
| 25 | Denmark | Dansk Selskab for Almen Medicin | DSAM | <http://www.dsam.dk/flx/english> | 1 March 2015 | Excluded since it does not provide publicly accessible document on guidelines development or COI issue guideline related | Small part of the website in English |
| 26 | Denmark | Sundhedsstyrelsen | SHS | http://sundhedsstyrelsen.dk/en/ | 15 December 2015 | Excluded since it does not provide publicly accessible document on guidelines development or COI issue guideline related | http://sundhedsstyrelsen.dk/en/about-us/targets-and-tasks/conflicts-of-interests |
| 27 | Estonia | University of Tartu Medical Faculty, Estonian Health Insurance Foundation, World Health Organization | HE-TU | http://www.ut.ee/en | 17 June 2015 | Included. Full text of document assessed for eligibility | Estonian handbook for guidelines development. 2011  http://www.ut.ee/en/kontakt/arstiteaduskondla  http://whqlibdoc.who.int/publications/2011/9789241502429_eng.pdf |
| 28 | Finland | Current care guidelines/The Finnish Medical Society Duodecim | FMSD | http://www.duodecim.fi/english | 17 June 2015 | Included. Full text of document assessed for eligibility | http://kaypahoito.fi/web/english/home  http://www.ebm-guidelines.com/dtk/ebmg/home/process description  http://www.terveysportti.fi/xmedia/ccs/process/Suositus.html (process development)  http://www.terveysportti.fi/xmedia/ccs/process/Sidonnaisuudet.html  Suomalainen Lääkäriseura Duodecim  http://www.duodecim.fi/english/products/current-care-guidelines/ |
| 29 | Finland | Duodecim Medical Publications ltd | KD | http://www.duodecim.fi/kustannus/ | 17 June 2015 | Excluded since it is not a guideline developer |  |
| 30 | France | Haute Autorité de Santé | HAS | http://www.has-sante.fr/portail/ | 17 June 2015 | Included. Full text of document assessed for eligibility | http://www.has-sante.fr/portail/upload/docs/application/pdf/2011-01/guide_methodologique_recommandations_pour_la_pratique_clinique.pdf  Haute Autorité de Santé Guide des déclarations d’intérêts et de gestion des conflits d’intérêts. Guide de prévention et de gestion des conflits d’intérêts adopté par le Collège le 24 juillet 2013 Available <http://www.has-sante.fr/portail/upload/docs/application/pdf/guide_dpi.pdf>  <http://www.has-sante.fr/portail/upload/docs/application/pdf/guide_dpi.pdf> (DOI) |
| 31 | Germany | Arbeitsgemeinschaft der Wissenschaftlichen Medizinischen Fachgesellschaften (German Association of the Scientific Medical Societies) | AWMF | http://www.awmf.org/en | 17 June 2015 | Included. Full text of document assessed for eligibility | http://www.awmf.org/leitlinien/awmf-regelwerk/awmf-guidance.html  http://www.awmf.org/fileadmin/user_upload/Leitlinien/AWMF-Regelwerk/AWMF-Guidance_2013.pdf |
| 32 | Germany | German Agency  for Quality in Medicine  (Das Ärztliche Zentrum für Qualität in der Medizin) | AQuMed-ÄZQ | http://www.aezq.de/front-page-en?set_language=en | 25 May 2015 | Included. Full text of document assessed for eligibility | German Medical Association (GMA), National Association of Statutory Health Insurance Physicians (NASHIP), Association of the Scientific Medical Societies (AWMF). National Program for Disease Management Guidelines. Method Report. 4th edition. 2010. Available from: http://www.leitlinien.de/mdb/downloads/nvl/methodik/mr-engl-aufl-4-version-1.pdf |
| 33 | Germany | Deutsche Krebsgesellschaft | DKG | https://www.krebsgesellschaft.de/german-cancer-society.html |  | Excluded since it is on specific health topics/specialties |  |
| 34 | Germany | Zentrum Zahnärztliche Qualität | ZZQ | http://www.zzq-berlin.de/english/index.htm |  | Excluded since it is on specific health topics/specialties |  |
| 35 | Hungary | National Institute for Quality and Organizational Development in Healthcare and Medicines  (Állami Egészségügyi Ellátó Központ) | GYEMSZI | http://www.gyemszi.hu/aeek/ | 9 April 2015 | Excluded since it does not provide documents in English, French, Italian, Spanish |  |
| 36 | Hungary | National Advisory Board of Health Care | NABHC |  |  | Excluded since website not found or not accessible |  |
| 37 | International | European Society of Cardiology | ESC | https://www.escardio.org/ |  | Excluded since it is on specific health topics/specialties |  |
| 38 | International | Central Asian Network of EBM Centers (Kazakhstan, Kyrgyzstan, Uzbekistan, Tajikistan, Turkmenistan) | CAREBMC Net | http://www.carebms.net | 9 April 2015 | Excluded since website not found or not accessible |  |
| 39 | Intergovernmental | World Health Organization | WHO | http://www.who.int | 17 June2015 | Included. Full text of document assessed for eligibility | WHO handbook for guideline development (2014)  http://www.who.int/kms/handbook_2nd_ed.pdf |
| 40 | Ireland | The National Clinical Effectiveness Committee | NCEC | http://health.gov.ie/patient-safety/ncec/governance-ncec/ | 17 June 2015 | Included. Full text of document assessed for eligibility | Manual:  http://health.gov.ie/wp-content/uploads/2015/01/ncec_guideline_development_manual_january13.pdf  NCEC and conflict of interest policy:  http://health.gov.ie/patient-safety/ncec/governance-ncec/  http://health.gov.ie/patient-safety/ncec/resources-and-learning/ncec-processes-and-templates/ |
| 41 | Ireland | Mental Health Commission | MHC | http://www.mhcirl.ie/ |  | Excluded since it is on specific health topics/specialties |  |
| 42 | Italy | Sistema Nazionale Linee Guida | SNLG | http://www.snlg-iss.it | 25 June 2015 | Included. Full text of document assessed for eligibility | http://www.snlg-iss.it/cms/files/Manuale_PNLG_0.pdf |
| 43 | Kazakhstan | Republic Centre for Healthcare Development | RCHD |  |  | Excluded since website not found or not accessible |  |
| 44 | Korea | Korean Academy of Medical Science | KAMS | http://www.kams.or.kr/eng/ |  | Excluded since it does not provide publicly accessible document on guidelines development or COI issue guideline related |  |
| 45 | Lithuania | Lietuvos Respublikos Sveikatos Apsaugos Ministerija | LRSAM | http://sam.lrv.lt/lt/ | 9 April 2015 | Excluded since it does not provide documents in English, French, Italian, Spanish | The document that describes the development process in Lithuanian |
| 46 | Luxemburg | Conseil Scientifique du Domaine de la Santé | CSDS | http://www.conseil-scientifique.lu/ | 17 June 2015 | Included. Full text of document assessed for eligibility | Procédures pour l’établissement et la diffusion de référentiels de  bonne pratique par le Conseil Scientifique  <http://www.conseil-scientifique.lu/index.php?id=27> |
| 47 | Malaysia | Kementerian Kesihatan Malaysia | KKM | http://www.moh.gov.my/ | 9 April 2015 | Excluded since it does not provide documents in English, French, Italian, Spanish |  |
| 48 | Malta | Malta Department  of Medicine at Central Hospital Mater Dei | CHMD | https://ehealth.gov.mt/HealthPortal/health_institutions/hospital_services/mater_dei_hospital/mater_dei.aspx | 9 April 2015 | Excluded since it does not provide publicly accessible document on guidelines development or COI issue guideline related |  |
| 49 | Mexico | Centro National de Excelentia Tecnólogica en Salud | CENETEC | http://www.cenetec.salud.gob.mx | 17 June 2015 | Included. Full text of document assessed for eligibility | http://www.cenetec.salud.gob.mx/descargas/gpc/METODOLOGIA_GPC.pdf |
| 50 | Netherland | Dutch Institute for Healthcare Improvement | CBO | <http://www.cbo.nl> | 9 April 2015 | Excluded since it does not provide publicly accessible document on guidelines development or COI issue guideline related |  |
| 51 | Netherland | Nederlands Huisarten Genootschap | NHG | https://www.nhg.org/dutch-college-general-practitioners | 9 April 2015 | Excluded since it does not provide publicly accessible document on guidelines development or COI issue guideline related |  |
| 52 | Netherland | Regieraad Kwaliteit van Zorg | RKZ | https://www.regieraad.nl | 9 April 2015 | Excluded since website not found or not accessible |  |
| 53 | Netherland | Rijksinstituut voor Volksgezondheid en Milieu | RIVM | http://www.rivm.nl/en |  | Excluded sinceit is on specific health topics/specialties |  |
| 54 | Netherland | Trimbos Instituut | TI | http://www.trimbos.org/ |  | Excluded since it is on specific health topics/specialties |  |
| 55 | Netherland | Integraal Kankercentrum Nederland | IKNL | https://www.iknl.nl/over-iknl/about-iknl |  | Excluded since it is on specific health topics/specialties |  |
| 56 | Netherland | Koninklijk Nederlands Genootschap voor Fysiotherapie | KNGF | https://www.fysionet-evidencebased.nl/index.php/kngf-guidelines-in-english |  | Excluded since it is on specific health topics/specialties |  |
| 57 | Netherland | Landelijk Expertisecentrum Verpleging and Verzorging | LEVV | http://www.levv.nl |  | Excluded since it is on specific health topics/specialties |  |
| 58 | New Zealand | New Zealand Guidelines Group | NZGG | http://www.health.govt.nz/ | 9 April 2015 | Excluded since it is not a guideline developer | Went in liquidation in mid-2012 |
| 59 | Norway | Helsedirektoratet | HD | https://helsedirektoratet.no/english | 9 April 2015 | Excluded since it does not provide publicly accessible document on guidelines development or COI issue guideline related |  |
| 60 | Peru | Ministerio de Salud | MS | http://www.minsa.gob.pe/ | 17 June 2015 | Included. Full text of document assessed for eligibility | Norma técnica para la elaboratión de Guías de Práctica Clínica. 2006  http://bvs.minsa.gob.pe/local/MINSA/1176_DGSP196.pdf |
| 61 | Portugal | Centro de Estudos de Medicina Baseada na Evidência, Faculdade de Medicina, Universidade de Lisboa | CEMBE | http://www.medicina.ulisboa.pt/investigacao/unidades-de-investigacao-autonomas/cembe-en/ | 9 April 2015 | Excluded since it does not provide publicly accessible document on guidelines development or COI issue guideline related |  |
| 62 | Portugal | [Direção-Geral da Saúde](https://www.dgs.pt/) | DGS | https://www.dgs.pt/directorate-general-of-health.aspx | 9 April 2015 | Excluded since it does not provide publicly accessible document on guidelines development or COI issue guideline related |  |
| 63 | Portugal | Direção nacional da Associação Portuguesa de Medicina Geral e Familiar | APMGF | http://www.apmgf.pt/ | 9 April 2015 | Excluded since it does not provide documents in English, French, Italian, Spanish |  |
| 64 | Romania | Centrul National de Studii pentru Medicina Familiei | CNSMF | http://cnsmf.ro/ | 9 April 2015 | Excluded since it does not provide documents in English, French, Italian, Spanish |  |
| 65 | Romania | Ministerul Sănătătii | MSA | http://www.ms.ro/ | 9 April 2015 | Excluded since it does not provide documents in English, French, Italian, Spanish |  |
| 66 | Saudi Arabia | King Saud bin Abdulaziz University for Health Sciences, National and Gulf Center for Evidence Based Health Practice | EBHP | http://ngcebm.ksau-hs.edu.sa/ | 17 June 2015 | Included. Full text of document assessed for eligibility. | Guidance for Clinical Practice Guideline Development. Adaptation and Endorsement. 2008  http://ngcebm.ksau-hs.edu.sa/images/content/NGCEBHC-Draft-3-CPG-Guidance.pdf |
| 67 | Slovakia | Národný Inštitút Kvality a Inovácií (National Institute of Quality and Innovation) | NIKI | http://www.quality.healthnet.sk/ | 17 June 2015 | Included. Full text of document assessed for eligibility | A guideline developers’ handbook. National institute of quality and innovations. October 2005 http://www.quality.healthnet.sk/EBM/Guidelines_development_handbook_Slovak_version_v1.pdf |
| 68 | Slovenia | Ministrstvo za Zdravje | MZ | http://www.mz.gov.si/ | 17 June 2015 | Excluded since it does not provide documents in English, French, Italian, Spanish |  |
| 69 | Spain | GuíaSalud | GS | http://portal.guiasalud.es/ | 17 June 2015 | Included. Full text of document assessed for eligibility | Grupo de trabajo sobre GPC (2007). Elaboración de Guías de Práctica Clínica en el Sistema Nacional de Salud. Manual Metodológico. Madrid: Plan Nacional para el SNS del MSC. Instituto Aragonés de Ciencias de la Salud-I+CS; 2007. Guías de Práctica Clínica en el SNS: I+CS Nº 2006/0I. -%20Elaboracion%20GPC%20en%20el%20SNS.pdf  http://www.guiasalud.es/emanuales/elaboracion/index-02.html |
| 70 | Spain | Osakidetza | OSTEBA | http://www.osakidetza.euskadi.eus | 9 April 2015 | Excluded since it does not provide publicly accessible document on guidelines development or COI issue guideline related |  |
| 71 | Spain | Redeguias-Spanish Network for Research on Guidelines | RG |  |  | Excluded since website not found or not accessible |  |
| 72 | Sweden | National Board of Health and Welfare  (Socialstyrelsen) | SS | http://www.socialstyrelsen.se/english | 17 June 2015 | Included. Full text of document assessed for eligibility | <http://www.socialstyrelsen.se/nationalguidelines/howwedrawuptheguidelines> |
| 73 | Switzerland | Centre d’épidémiologie Clinique at  Institut universitaire de médecine sociale et préventive, Lausanne | CEPIC | <http://www.chuv.ch/cepic/ac_qui.htm> | 9 April 2015 | Excluded since it does not provide publicly accessible document on guidelines development or COI issue guideline related |  |
| 74 | Ukraine | The State Expert Center, Ministry of Health | SEC | http://www.moz.gov.ua/ua/portal/ | 9 April 2015 | Excluded since it does not provide documents in English, French, Italian, Spanish |  |
| 75 | United Kingdom-Scotland | Scottish Intercollegiate Guidelines Network | SIGN | http://www.sign.ac.uk/ | 17 June 2015 | Included. Full text of document assessed for eligibility | <http://www.sign.ac.uk/pdf/doi-policy.pdf>  Scottish Intercollegiate Guidelines Network. SIGN 50 - A guideline developer’s handbook; 2014.  Available: http://www.sign.ac.uk/pdf/sign50.pdf |
| 76 | United Kingdom-England | National Institute for Health and Care Excellence | NICE | http://www.nice.org.uk/ | 17 June 2015 | Included. Full text of document assessed for eligibility | <http://www.nice.org.uk/article/pmg20/resources/non-guidance-developing-nice-guidelines-the-manual-pdf>  <https://www.nice.org.uk/Media/Default/About/Who-we-are/Policies-and-procedures/Code-of-practice-for-declaring-and-managing-conflicts-of-interest.pdf> |
| 77 | United States | Agency for Healthcare Research and Quality | AHRQ | http://www.ahrq.gov/ | 9 April 2015 | Excluded since it is not a guidelinedeveloper |  |
| 78 | United States | American Academy  of Neurology | AAN | https://www.aan.com/ |  | Excluded since it is on specific health topics/specialties |  |
| 79 | United States | American Academy  of Otolaryngology-Head and Neck Surgery | AAO | http://www.entnet.org/ |  | Excluded since it is on specific health topics/specialties |  |
| 80 | United States | American College  of Cardiology | ACC | http://www.acc.org/ |  | Excluded since it is on specific health topics/specialties |  |
| 81 | United States | American College  of Chest Physicians | ACCP | http://www.chestnet.org/ |  | Excluded it is on specific health topics/specialties |  |
| 82 | United States | American College  of Physicians | ACP | https://www.acponline.org/ | 15 June 2016 | Included. Full text of document assessed for eligibility | <http://www.acponline.org/clinical_information/guidelines/> <https://www.acponline.org/clinical_information/guidelines/guidelines/conflicts_cgc.htmreaccessed>  <http://annals.org/article.aspx?articleid=745942> |
| 83 | United States | American Gastroenterological Association | AGA | http://www.gastro.org/ |  | Excluded since it is on specific health topics/specialties |  |
| 84 | United States | American Psychological Association | APA | http://www.apa.org/ |  | Excluded since it is on specific health topics/specialties |  |
| 85 | United States | American Society  of Clinical Oncology | ASCO | http://www.asco.org/ |  | Excluded since it is on specific health topics/specialties |  |
| 86 | United States | American Society  for Radiation Oncology | ASTRO | https://www.astro.org/ |  | Excluded since it is on specific health topics/specialties |  |
| 87 | United States | US Centers for Disease Control and Prevention-Advisory Committee on Immunization Practices | CDC-ACIP | http://www.cdc.gov/vaccines/acip/recs/grade/about-grade.html | 17 June 2015 | Included. Full text of document assessed for eligibility | Ahmed F, Temte JL, Campos-Outcalt D, Schünemann HJ, ACIP Evidence Based  Recommendations Work Group (EBRWG). Methods for developing evidence-based  recommendations by the Advisory Committee on Immunization Practices (ACIP) of the  U.S. Centers for Disease Control and Prevention (CDC). Vaccine. 2011 Nov  15;29(49):9171-6 available http://www.cdc.gov/vaccines/acip/recs/grade/about-grade.html  Ahmed F. U.S. Advisory Committee on Immunization Practices Handbook for Developing Evidence-based Recommendations. Version 1.2. Atlanta, GA: Centers for Disease Control and Prevention (CDC); 2013. http://www.cdc.gov/vaccines/acip/recs/GRADE/about-grade.html#resources  Citation of role of Committee available at http://www.cdc.gov/vaccines/acip/committee/index.html which include article Smith JC, Snider DE, Pickering LK. Immunization Policy Development in the United States: The Role of the Advisory Committee on Immunization Practices. Ann Intern Med. 2009;150:45-49. doi:10.7326/0003-4819-150-1-200901060- |
| 88 | United States | US Centers for Disease Control and Prevention- Healthcare Infection Control Practices Advisory Committee | CDC-HICPAC | http://www.cdc.gov/hicpac/about.html | 17 June 2015 | Included. Full text of document assessed for eligibility | http://www.cdc.gov/hicpac/guidelineMethod/guidelineMethod.html |
| 89 | United States | Center for International Rehabilitation | CIR | <http://www.cirnetwork.org/> |  | Excluded since it is on specific health topics/specialties |  |
| 90 | United States | Infectious Diseases Society of America | IDSA | http://www.idsociety.org/ |  | Excluded it is on specific health topics/specialties |  |
| 91 | United States | Kaiser Permanente | KP | https://healthy.kaiserpermanente.org/ | 17 June 2015 | Included. Full text of document assessed for eligibility | Davino-Ramaya C, Krause LK, Robbins CW, Harris JS, Koster M, Chan W, Tom GI. Transparency matters: Kaiser Permanente's National Guideline Program methodological processes. Perm J. 2012 Winter;16(1):55-62.  http://www.kpihp.org/wp-content/uploads/2013/07/KPStories-v2n2-ClinicalGuidelines-FINAL.pdf  Kaiser Permanente National Guideline Program Process and Methodology for Systematic Development of Clinical Practice Recommendations (monograph on the Intranet).  Oakland, CA: Kaiser Permanente; 2011 Mar 15. [cited 2012 Feb 8].  Available from: <https://wiki.kp.org/wiki/download/attachments/102107054/NGP+Methodology+3-15-11.pdf?version=1&modificationDate=1305579496000>; password protected. |
| 92 | United States | US Preventive Services Task Force | USPSTF | http://www.uspreventiveservicestaskforce.org/ | 17 June 2015 | Included. Full text of document assessed for eligibility | U.S. Preventive Services Task Force Procedure manual. AHRQ publication NO.08-05118-EF March 2014  http://www.uspreventiveservicestaskforce.org/Page/Name/methods-and-processes  http://www.uspreventiveservicestaskforce.org/Page/Name/methods-and-processes |
